# Automatic Detection of Cognitive Impairment with Virtual Reality

**DOI:** 10.3390/s23021026

**Published:** 2023-01-16

**Authors:** Farzana A. Mannan, Lilla A. Porffy, Dan W. Joyce, Sukhwinder S. Shergill, Oya Celiktutan

**Affiliations:** 1 Department of Engineering, King’s College London, London WC2R 2LS, UK; 2 Department of Psychosis Studies, Institute of Psychiatry, Psychology and Neuroscience, King’s College London, London SE5 8AF, UK; 3 Department of Primary Care and Mental Health, Institute of Population Health, University of Liverpool, Liverpool L69 3BX, UK; 4 Kent and Medway Medical School, Canterbury CT2 7NZ, UK

**Keywords:** feature engineering, linear regression, statistical learning, psychosis, cognitive assessment, virtual reality

## Abstract

Cognitive impairment features in neuropsychiatric conditions and when undiagnosed can have a severe impact on the affected individual’s safety and ability to perform daily tasks. Virtual Reality (VR) systems are increasingly being explored for the recognition, diagnosis and treatment of cognitive impairment. In this paper, we describe novel VR-derived measures of cognitive performance and show their correspondence with clinically-validated cognitive performance measures. We use an immersive VR environment called VStore where participants complete a simulated supermarket shopping task. People with psychosis (k=26) and non-patient controls (k=128) participated in the study, spanning ages 20–79 years. The individuals were split into two cohorts, a homogeneous non-patient cohort (k=99 non-patient participants) and a heterogeneous cohort (k=26 patients, k=29 non-patient participants). Participants’ spatio-temporal behaviour in VStore is used to extract four features, namely, route optimality score, proportional distance score, execution error score, and hesitation score using the Traveling Salesman Problem and explore-exploit decision mathematics. These extracted features are mapped to seven validated cognitive performance scores, via linear regression models. The most statistically important feature is found to be the hesitation score. When combined with the remaining extracted features, the multiple linear regression model resulted in statistically significant results with $R^{2}$ = 0.369, F-Stat = 7.158, p(F-Stat) = 0.000128.

## 1. Introduction

Cognitive impairment in neuropsychiatric disorders are well documented [1] and difficult to treat [2]. Detection of cognitive impairment requires validated psychometric tests which range in sensitivity and specificity (to detect deviations from the healthy population norms) and in terms of coverage of signs/symptoms relevant to patients’ experience. In addition, short clinical (or “bedside”) screening tests (e.g., [3,4]) are generally quick to administer but lack specificity and have limited coverage of a subset of cognitive domains, whereas very detailed test batteries have broader coverage but can take up to 90 min to administer [5,6].

To address the gaps in cognitive assessment methods, there has been increasing effort to use ICT (Information and Communications Technologies) [7]. The virtual reality (VR) methods currently explored show potential for improvement and innovation on clinical cognitive assessment. Patients presenting cognitive impairment exhibit reduced function of core cognitive domains including working memory, verbal learning, and other executive functions (e.g., prioritising, organising, and impulse control). Two cognitive domains integral to this study are spatio-temporal capacity and short term memory. The former is particularly difficult to measure at present but is well captured in VR environments. VR systems offer ecological validity (ability to predict real-life behaviour) [7], construct validity (ability of VR methods to measure what they aim to—cognition) [8], and a means of early detection [9]. Our study takes VR’s potentials further by quantifying qualitative behavioural presentations of cognitive impairment. This study contributes features based on distance, reaction time, and strategy. Finally, it presents potential for capturing cognition on the continuum it truly spans through an ordinary least-squares multiple linear regression model.

This paper introduces four novel features to quantify sensitive changes in patient behaviour, mapping these to clinically accepted cognitive scores by immersing participants in a real-life activity (e.g., shopping) using VR. For this purpose, we use VStore, a VR environment co-designed by Vitae VR and the Institute of Psychiatry, Psychology and Neuroscience at King’s College London, United Kingdom, and the study designed, and data collected in [10]. The VR immerses patients into a simulation supermarket and tasks them with completing a number of relevant daily activities where symptoms would present. We focus on patients with Psychosis (Schizophrenia) who have well described cognitive deficits. Psychosis is a severe and enduring mental illness with a phenotype that typically presents in adulthood. It is marked by symptoms such as hallucinations and cognitive impairment. In turn, the condition is associated with loss of capacity central to daily functioning [11].

This research addresses several key questions. Firstly, whether the extent of cognitive impairment can reliably be predicted on a continuum from patient behaviour in a VR environment. This is something that has not been explored before. If this is achievable, to quantify the corresponding key features to be extracted from data captured in such an environment. This can be attained if drivers of subject behaviour are well understood and meaningfully mapped to their presentations in the data captured. Extracted features must be modelled on measures of deviation from some optimal behavioural performance to index cognitive status. The major contribution of this work is defining and extracting four features that quantitatively measure behavioural markers of cognition. Moreover, that these features have been combined into a predictive model in turn mapping extent of cognitive impairment on the continuum it truly spans. The four features are route optimality score, proportional distance score, execution error score, and hesitation score. The features employ the Traveling Salesman Problem and explore-exploit decision mathematics. This study maps the extracted features to standard cognitive assessments of attention and memory (e.g., detection, one card learning, one back card, identification), via ordinary least-squares multiple linear regression models. The most statistically significant feature was found to be the hesitation score against cognitive predictor detection (DET). Hesitation score alone performed up to two orders of magnitude greater in statistical significance than all four features combined.

## 2. Related Work

Standard cognitive assessments are often divorced from functional capacity, which refers to ability to perform essential daily life tasks. Traditionally in medicine, these cognitive domains are measured using qualitative pen and paper methods. This study demonstrates the added value of sensitivity and measure of extent of impairment that a VR, statistical learning based cognitive assessment offers neuroscience. The following literature was explored to understand the evolution of cognitive assessments towards measures of functional cognition that leverage VR. It also considers gaps in literature that this study bridges in quantifying qualitative behaviours and predicting cognition on a continuum.

### 2.1. Pen and Paper Methods

The Mini-Mental State Examination (MMSE) is built on cognitive state tests [12]. This is a 30 point test categorised under a participant’s attention, recall, language, and ability to copy. Based on their cognitive performance the participant is ranked as either no impairment, mild impairment, or severe impairment [13]. There are a number of advantages to this test. As a well-adopted and established method, there is sufficient data available to draw reliable (reproducible under the same conditions) conclusions from. The mini-tests are also short and low complexity to carry out. The accuracy, precision and range of cognitive function assessed makes this a popular choice for physicians [14]. MMSE requires comprehension of language, creating a bias on overall language ability—independent of cognitive ability and is biased by educational attainment. MMSE does not assess visuo-spatial reasoning to the same extent as it does other cognitive facets [15]. This is something the VStore environment addresses implementing VR and in turn measuring spatio-temporal, six-axis motion (*x*, *y*, *z*, yaw, pitch, roll), of the participant.

The clock-drawing test is another pen and paper means of cognitive assessment [16]. It requires the participant to write the numbers onto a blank clock face drawing and then draw out the arms of the clock to correctly indicate a time [16]. Again, a scoring of cognition is made based on ability to carry out the different parts of the assessment. It is implemented as a fast screening and is an easy alternative for non-compliant patients [17]. The test excels in visuo-spatial reasoning assessment, where MMSE falls short. It is however unconstrained as a participant can draw something unexpected in response to the prompts that scoring does not account for. This test presents bias in favour of education and provides no assessment of language [18]. VStore addresses this in that participants have to understand the shopping list—through hearing it, and/or reading it before proceeding. Equally, the level of education is less likely to bias performance on such VR spatial tasks and multi-modal presentation of stimuli used in the task.

### 2.2. VR Cognitive Assessments

VR addresses the lack of large-scale spatial navigational assessment in pen-and-paper methods through multi-dimesional spatial reasoning whilst also assessing allocentric and egocentric navigation. The former being navigation with respect to a person’s surroundings and the latter to themselves. Cognitive impairment can naturally present in real-life situations that VR can simulate [19]. Participants with cognitive problems present difficulties on navigational parameters characteristic of conditions such as Alzheimer’s Disease (AD) and Psychosis. Another consideration in navigation is navigation memory also well assessed using VR [20]. This study successfully monitored and tested vehicular exploration of a city assessing both forms of navigation and their recollection. It specially focused on cognitive decline and could be similarly be considered for cases of cognitive impairment. Cognitive impairment reduces ability to carry out daily self-care tasks, VR assessments can simulate these tasks introducing varying complexity that pen and paper methods cannot [21]. One matter of concern with VR is a participant’s command of technology particularly in assessing generations that did not grow up with it.

A notable study [9] corroborates the finding that VR can identify cognitive problems more accurately than gold standard cognitive tests. In this study, the focus is on early onset AD and cognitive decline as opposed to cognitive impairment observed in Psychosis. Participants in this study traverse a path and listen for audio cues to stimulate reactions indicative of symptoms of cognitive decline. The measure is of absolute distance subverted from the path participants are tasked to follow. They found absolute distance error to be statistically significant (i.e., when assessed with Null Hypothesis significance testing at α=0.01). VStore embeds not only measures of subversion from optimal paths in the features extracted but also time-bound and strategic scores capturing executive function.

Another study [22] explores VR to rehabilitate concussed jockeys who have been injured subsequently facing cognitive impairment. It immerses recovering patients into a VR simulation where they are horse racing with visual cues to react with joy sticks capturing attention and decision making in the environment—medical markers of cognition. This study is ecologically valid for these participants and specific to this particular use-case. It measures reaction time through a number of means including monitoring eye movement. Reaction time is an accepted measure of cognitive capacity. Similarly, VStore and the features extracted innovate on how to measure medically accepted markers of behaviour and executive function. The features extracted from VStore go further in quantifying extent of cognitive impairment on a continuum.

The VStore study focuses on patients with cognitive impairment (Psychosis) rather than cognitive decline (e.g., AD). Its findings show potential for future work with patients experiencing cognitive decline.

### 2.3. Cognitive State Features

In previous literature, extracted features include reaction time, distance covered by participants, and accuracy of memory recall. Our study quantifies qualitative behavioural presentations of cognition with features centred on reaction time (hesitation score), distance covered by participants (proportional distance score), accuracy of execution (execution error score), and strategic decision making (hesitation score and route optimality score).

Current research cites works to measure cognition on a continuum by combining qualitative and discrete results into a predictive model. However, the research does not employ new or continuous features for measuring cognition in such modelling. One study uses subjective discrete self-scoring mood as an input to the model to generate a continuum by combining such qualitative or discrete markers into a predictive model [23]. Our study progresses on this by combining stand-alone continuous quantitative features into predictive models. The predictive models further map extent of cognitive impairment on a continuum.

A study in this field [24] combined six known physical health indicators into a regression model to predict cognitive function and potential decline. Health indicators included the six-minute walk test, cardiovascular risk and self scored mood test. This model had an explained variance of $R^{2}=0.37$ when using one variable in a regression and $R^{2}=0.47$ when using five features via cross validation [24]. Another study published in 2021 combined the aforementioned Mini-Mental State Examination (MMSE) scores into a predictive model mapping to Alzheimer’s Disease stating its best predictive model statistic to be $R^{2}=0.388$ (std. 0.073) [25]. Our study progresses on this by defining and extracting quantitative features that map to cognition rather than employing a predictive model based on existing pen and paper results.

## 3. VR Study

Early diagnosis of cognitive impairment can improve prognosis and remains a challenge for clinicians. This study addresses ongoing barriers to detecting early onset cognitive impairment through: (1) a behavioural-based VR assessment centred on daily activity, and (2) a quantitative, measure of extent of cognitive impairment on a continuum determined by features extracted from data in this environment.

In this section, we describe the VR environment used in our paper, VStore, and the clinical study to assess cognitive impairment.

### 3.1. VStore

**Figure 1:**
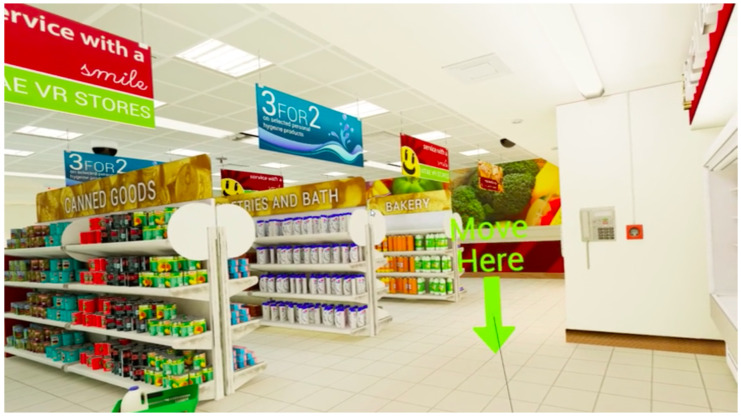
A snapshot from VStore from the perspective of a participant navigating. This frame presents a critical decision point in the VR supermarket where a participant has visibility of six items in the n-item (n=12) shopping list. Employing the classical traveling salesman problem (TSP) requires visibility of all locations to travel to ahead of starting the journey. At this location in VStore, the participant enters a six-point TSP that they must decide how to approach by taking into consideration that half of the items on the shopping list are visible.

VStore is a VR environment co-designed by Vitae VR and the Institute of Psychiatry, Psychology and Neuroscience, at King’s College London, United Kingdom [26]. The VR immerses patients into a simulation supermarket and tasks them with completing a number of daily activities centred on visiting a supermarket where symptoms would present. These tasks include collecting items from a shopping list, paying for shopping at a self-service checkout, and ordering a coffee. Timestamped participant movements were monitored across both translational and rotational degrees of freedom as they executed these tasks. A snapshot from VStore is given in Figure 1.

### 3.2. Clinical Study

The 154 participants included in the study were divided into 2 cohorts. Cohort 1 was a homogeneous cohort with non-patient participants only. Cohort 2 was heterogeneous with a mixture of non-patient and patient participants. The first cohort of participants was a baseline non-patient group used to test the extracted features. This homogeneity left minimal room for confounding variables to impact assessment of the computational method itself. Once there was confidence in this, the second cohort was applied to the computational method.

### 3.3. Procedure

Cohort 1 consists of 99 non-patient participants spanning ages 20–79 years (μ=40±17). Cohort 2 consists of 29 non-patient participants and 26 patients with psychosis spanning ages 20–79 years (μ=48±17). Participants across both cohorts were asked questions to ascertain individual frequency, comfort, and ability in technology use [10].

The clinical study included 28 male and female patients aged 18 to 60 with schizophrenia or a related psychotic-spectrum disorder, of these, 26 were included in our study. Participants were recruited from outpatient services at the South London and Maudsley (SLaM) National Health Service Foundation Trust. Eligible participants had to be stable on their antipsychotic medication for at least one month, and able to provide informed consent. Patients with an Axis I disorder other than psychosis (DSM-5), moderate to severe alcohol and/or substance use disorder, a history of severe motion sickness, a neurological illness, IQ below 70, mobility issues, and who are pregnant were excluded. Further exclusions in data included participants facing technical issues, failing the completion criteria for VStore, and extreme outliers in the dataset [27].

The clinical study took approximately 30 min to complete, including orientation, instructions, practice, and assessment. The assessment was set in a virtual supermarket holding 66 items under 9 item classifications across 4 shopping aisles, 1 set of fridges, and a section of fruit and vegetable stalls. In addition, there were self-checkouts and a coffee shop at the back of the shop floor. Participants were read out 12 items from a shopping list by an avatar at the entrance then tasked with memorising and recalling as many items from this list as possible. Following recall, participants were presented with the shopping list, including all 12 items, and instructed to navigate the virtual supermarket in collecting all items as quickly and accurately as possible.

Cognitive scores were collected from patient and non-patient cohorts immersed in the VStore simulation. Of the cognitive score, three were present in the outcome of reaction time: (1) measuring information processing speed (DET, detection); (2) measuring attention and working memory (ONB, one back task); and (3) measuring visual attention and vigilance (IDN, identification). There is a score measuring visual learning and short-term memory (OCL, one card learning) presents in the outcome of accuracy [28]. The final score cogstate composite aggregates all of these constituent scores into one overarching score.

Next, participants were tasked with carrying out a number of day-to-day activities where symptoms are likely to present—from collecting and paying for 12 items on a shopping list, to ordering and paying for a coffee. Timestamped participant motion across both translational and rotational degrees of freedom were measured throughout.

## 4. Proposed Methodology

The main objective of this paper is to devise a continuous scoring system for extent of cognitive impairment based on participant performance in a VR environment. We devised novel features extracted from time-stamped translational and rotational participant data. This invited feature extraction of scores centred on the Traveling Salesman Problem and the explore-exploit decision trade-offs.

### 4.1. Feature Extraction

In this section, we describe the features extracted to test the hypothesis that behaviours can be quantified in a data driven manner. VStore delivers continuous, highly granular spatio-temporal data. There is a trade-off to be made between handling of global and specific information where imbalance can lead to overfitting. Due to the changing fields of view experienced by a participant as they navigated VStore, it was problematic to treat the supermarket as a static map. Rather, it was meaningful to consider participant reactions to each frame of VStore they experienced as they navigated the environment.

Equally, it was important to devise features that account for this evolution. Upon trialling VStore as researchers, it became apparent that participant behaviour could be a meaningful focus for the features. To quantify these qualitative participant behaviours, the below key observations were considered in the context of VStore. The basis of these observations are agnostic of VStore and can be transferred to other VR environments.

1. VStore accounts for slow walkers as it is a VR simulation, one command to a controller is equivalent to one human stride. Hence pace of movement through this environment is better constrained to decision making as opposed to physical ability. This is one of the key advantages of VR cognitive assessments, they also enable large-scale spatial navigation to be assessed.
2. The virtual supermarket shop floor consists of four shopping aisles with further shelving spanning the perimeter of the store. In some positions of the VR environment, participants can see what is on many other shelves of the aisles without having to walk down an aisle to find them. This is particularly significant at the fridges where participants can see six items on the list at once. This stage of the VR environment can be modelled on Traveling Salesman Problem (TSP). Any VR based cognitive assessment that requires decision making between simultaneously visible landmarks can also be modelled on TSP. The participant paths taken may provide insight on executive function, specifically, goal-directed motor function.
3. Aisles are also labelled with the category of items they contain whether a participant can see the item or not, introducing bias to the aisles a participant explores. This is not simply an explore-exploit feedback system but one that invites strategic and optimal route planning, and cognition permitting. This observation can be translated to sophisticated VR cognitive assessments that compound behaviours in decisions made.

Inspired by these observations, we defined and extracted four features that account for critical behavioural presentations of cognition in VStore. These features are route optimality score, proportional distance score, execution error score, and hesitation score—described in sequel. The scores are centred on reaction time, distance covered, accuracy of execution and participant strategy.

#### 4.1.1. Spatial Features

There were two spatial features extracted using the Traveling Salesman Problem (TSP) algorithm in their definition: route optimality score and proportional distance score.

##### Route Optimality Score

**Figure 2:**
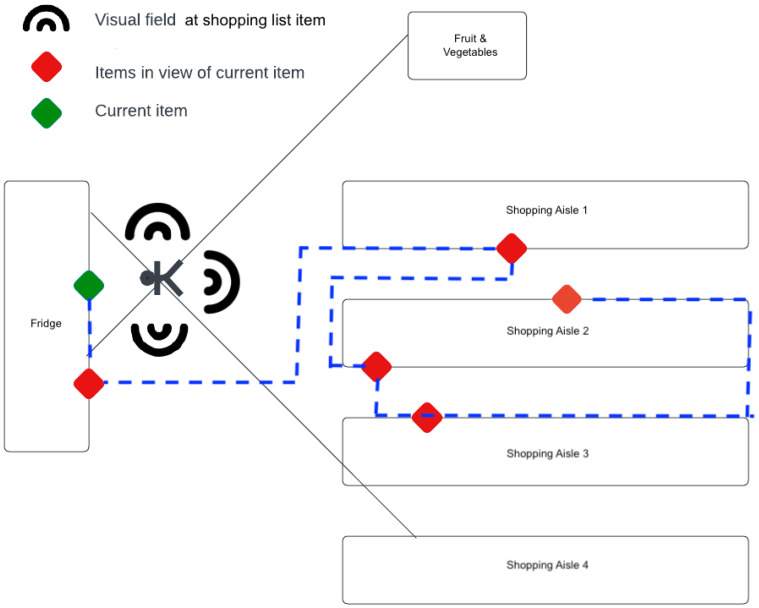
Schematic outlining rotational field of view and items included that hesitation score and spatial features employ. Blue path represents optimal solution to TSP. Please note that this figure is for illustrative purposes and does not reflect the actual proportions in the VStore environment.

**Figure 3:**
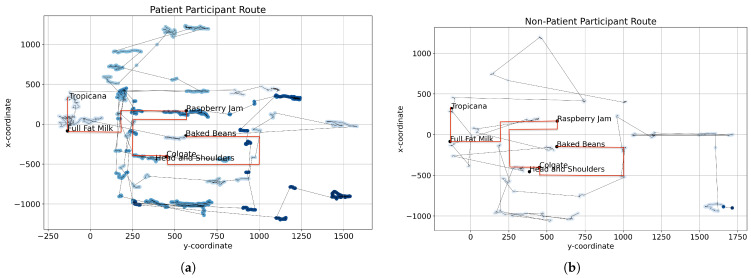
(**a**) Non-patient path taken where darker blue presents more time spent at a location and connections between nodes represent the path followed. Red is the optimal route for *n*-point (n=6) TSP. (**b**) Psychosis patient path taken where darker blue presents more time spent at a location and connections between nodes represent the path followed. Red is the optimal route for *n*-point (n=6) TSP.

The route optimality score aims to measure behavioural manifestations of executive function. This score was centred on solving a *n*-point TSP, where the n points represent the number of visible items in the shopping list when they reach the fridge in the supermarket (n=6 in our case). TSP is a classic NP-hard combinatorics problem [29] where a “salesman” must navigate a route visiting all “cities” subject to constraints, e.g., taking the shortest, most efficient possible route. This translates to the n items (nodes) on the shopping list the participant can see when reaching the fridge (see Figure 1) in the VStore environment. At this stage the participant must decide the optimal order for collecting these n visible items to reduce distance based cost of their overall route (see Figure 2). The route optimality score in turn captures any strategic decision-making at this critical stage in the environment. It accounts for the order that the participant chooses to collect these items, comparing this choice to the optimal TSP solution. The optimal TSP solution is mapped in the red route in Figure 3. The participant’s chosen order is then translated into an overall cost (by distance) attributed to optimally transitioning between the items in the order they collected them and compared against the baseline TSP solution for collecting these n items. This means of measuring participant performance was chosen as opposed to the true distance they covered to isolate participant strategy from their ability to execute a chosen strategy, which could be conflated with technology familiarity and other variables outside of cognitive markers.

In computing the optimal path, dynamic programming (DP) [30] was employed to an [x,y] mapping of the virtual supermarket shop floor, *n* items (n=6) to be collected and the obstacles presenting in the environment. DP guaranteed to find the distance-optimal solution to the TSP. However, its time complexity exponentially increases with the number of nodes so is only appropriate for a relatively small number of nodes (≤10) where nodes refer to the items in the shopping list in our case.

(1) $$\mathrm{Route}\;\mathrm{Optimality}\;\mathrm{Score}=\frac{\mathrm{Shortest}\;\mathrm{distance}\;\mathrm{given}\;\mathrm{participant}’s\;\mathrm{chosen}\;\mathrm{order}\;\mathrm{of}\;\mathrm{collection}}{\mathrm{Distance}\;\mathrm{of}\;\mathrm{TSP}\;\mathrm{solution}\;\mathrm{for}\;\mathrm{collecting}\;\mathrm{six}\;\mathrm{items}}$$

The standard assumption in TSP is that there are direct links between nodes (shopping items). Hence, in computing these links (paths taken between collecting items) we enforced the rule that the optimal routing could only employ movements perpendicular to the orthogonal axes defining the supermarket floor, accounting for obstacles in VStore, such as shopping aisles (see Figure 2). This defines relative distances (between the individual VStore participant and the six visible shopping items) as Manhattan ($L_{1}$ metric) distances. The complete graph of directly paired nodes (shopping items) and relative distance matrix were employed in the cost function for computing the optimal path. The cost was positive and symmetric based on the cost of traversing from one node to another in distance travelled. The only transition that does not hold is the transition from an item back to itself, such transitions were set to 0 in the distance matrix. In computing the next item to visit, the solver repeatedly optimised for edges of the graph with cheapest distance-based cost whilst keeping track of items already collected on the journey.

##### Proportional Distance Score

This score leans on TSP to extract the optimal path for completing the shopping list task by distance (collecting all 12 items). The distance covered by participants in completing the task on the path they chose to navigate the environment was taken as a proportion of this optimal path. Participants do not have full visibility of all items spanning the environment, in turn a score of 1 is highly unlikely. The purpose of this score is to standardise how close the participant was to the optimal TSP-derived route for the task rather than comparing non-patient to patient populations. The distance of the optimal route was taken from the TSP solution computed as per the route optimality score but for all 12 shopping list items rather than the 6 visible at the fridge (see Figure 1). This method is more robust for VR environments capturing cognitive impairment based on paths taken as it accounts for non-patient cohorts deviating from the most computationally optimal path as well as patient cohorts. This is a significant development on current VR assessment methods that do not compare performance of all participants to a baseline [9]. It is important to note that deviation of non-patient cohorts from a baseline standard can also present, owing to natural aging and other variables that comparing the two cohorts on absolute distance alone does not consider.

(2) $$\mathrm{Proportional}\;\mathrm{Distance}\;\mathrm{Score}=\frac{\mathrm{Distance}\;\mathrm{participant}\;\mathrm{covered}\;\mathrm{completing}\;\mathrm{VStore}}{\mathrm{Distance}\;\mathrm{of}\;\mathrm{TSP}\;\mathrm{solution}\;\mathrm{for}\;\mathrm{collecting}\;12\;\mathrm{items}}$$

#### 4.1.2. Execution Error Score

The execution error score captures the ability of the participant to collect the 12 items on the list. This score extracts all items collected by the participant whilst navigating the shopping list task including the 12 items on the full shopping list necessary to complete the shopping task in VStore. It then calculates this surplus as a proportion of the 12 necessary items and accounts for the error made compared to only collecting the 12 items on the shopping list. Cognitive processes leading to the difference between the items collected and the 12 stated can be indicative of executive function from ability to correctly identify items, to the ability to read and follow the shopping list. The concept of this feature can be translated to other VR cognitive assessments where the count of actions expected and instructed is compared against those carried out by participants.

(3) $$\mathrm{Execution}\;\mathrm{Error}\;\mathrm{Score}=1-\frac{\mathrm{Number}\;\mathrm{of}\;\mathrm{additional}\;\mathrm{items}\;\mathrm{collected}}{12\;\mathrm{items}\;\mathrm{on}\;\mathrm{shopping}\;\mathrm{list}}$$

Please note that the execution error score considers surplus items collected only in calculation as participants were excluded and considered to have failed VStore if unable to collect the required 12 items. If a participant correctly collected only the 12 items on the shopping list, their execution error score would be 1.

#### 4.1.3. Hesitation Score

Hesitation score is computed on reaction time and explore-exploit decisions with the changing visual field experienced through VStore as illustrated in Figure 2. Research has shown that reaction time is a medically recognised marker of cognitive capacity [31]. This paper validates the basis of this marker and the significance of more complex reaction time testing that combines different components of cognition with reaction time [31]. Hesitation score captures executive functioning of participants. It is a time-bound feature measuring the average time taken for a participant to pick up a shopping list item from the moment it first enters their visual field. In computing this feature, it was first confirmed that all participants had passed a visual acuity check to ensure assumptions of the hesitation score were clinically valid. In considering the evolving landscape of the VR supermarket, the participant continually evaluates a trade-off between exploration and exploitation in their decisions [31]. A depiction of the difference in hesitation is expressed in Figure 3 showing the difference in time spent at different [*x*,*y*] coordinate locations across the shop floor. The darker the blue spot, the longer the time spent in a location. The patient spent significantly more time at locations they visited than the non-patient. Moreover, the patient visited more locations and based on their trajectory between visitations, revisited locations multiple times.

The timestamps at which a participant picked items in the shopping list were extracted from their data log and used as a basis for calculating the relative times between picking all items in the shopping list. This was akin to the Manhattan distance matrix employed in the TSP based features.

(4) $$\mathrm{Hesitation}\;\mathrm{Score}=\frac{1}{12}\sum_{i=1}^{12}\frac{\mathrm{Time}\;\mathrm{taken}\;\mathrm{to}\;\mathrm{collect}\;\mathrm{item}\;i\;\mathrm{once}\;\mathrm{visible}}{\mathrm{Total}\;\mathrm{time}\;i\;\mathrm{is}\;\mathrm{visible}\;\mathrm{during}\;\mathrm{VStore}},$$

where total time *i* is visible during VStore is standardised to locations *i* is known to be visible and visited by all participants. This time does not account for any additional exposures to item *i* throughout VStore.

The mapping of the shop floor was used to identify the [x,y] locations of the items and those surrounding it. As shown in Figure 2, at each shopping list item location in VStore, a participant could rotate about their position to broaden their field of view extending it to other shopping list items. A maximum of 270 degrees visibility was assumed at any location where participant’s field of view was unobstructed by shopping aisles. At each of these n=12 locations, at most five other shopping list items were visible at any one shopping list item location, and at the least, no other shopping list items were visible. Hesitation score computes and averages the time it takes a participant to collect each item from the moment it first enters their field of view in proportion to the total time it is visible to them in VStore given these 12 locations. Hesitation score does not account for further exposures to the items given participant path traversed. It exclusively considers the 12 shopping list item locations in computation.

## 5. Cognitive Score Prediction

In this paper, we take into consideration the seven measures introduced in the clinical procedure section (see Section 3.3) that can be correlated to cognition. Of the seven measures in our study, five are cognitive battery scores, IQ, and age. Of these five cognitive battery scores three present as an outcome indexing reaction time: (1) DET, detection, measuring information processing speed; (2) ONB, one back task, measuring attention and working memory; and (3) IDN, identification, measuring visual attention and vigilance. The remaining two include OCL, one card learning, which is a score measuring visual learning and short-term memory and presents in the outcome of accuracy [28]. Finally, cogstate composite score aggregates the cognitive batteries into one overarching score.

We employed ordinary least-squares multiple linear regression to model the four extracted features (route optimality, proportional distance, execution error, and hesitation scores) against the cognitive predictors. When more than one extracted feature is modelled, this can estimate the values of model coefficients, in turn explaining the response of each cognitive predictor. The key statistics observed in evaluating multiple linear regression models are $R^{2}$, F-Stat, p(F-Stat), and t-values.

We built seven multiple linear regression models mapping the four engineered features against the cognitive predictors and estimated parameters using ordinary least-squares where $y_{0}\to y_{6}$ are the predictor scores and $\beta_{0}\to \beta_{3}$ are the regression coefficients.

We then modelled:

$$\begin{array}{cc} {\mathrm{Predictor}}_{\left(y_{0}\to y_{6}\right)}\; & =\mathrm{Constant}\\ \; & \;+\beta_{0}\left(\mathrm{Proportional}\;\mathrm{Distance}\;\mathrm{Score}\right)\\ \; & \;+\beta_{1}\left(\mathrm{Route}\;\mathrm{Optimality}\;\mathrm{Score}\right)\\ \; & \;+\beta_{2}(\mathrm{Execution}\;\mathrm{Error}\;\mathrm{Score})\\ \; & \;+\beta_{3}\left(\mathrm{Hesitation}\;\mathrm{Score}\right) \end{array}$$

where,

$$\begin{array}{c} y_{0}\to y_{6}=\left[\begin{array}{c} \mathrm{Age}\\ \mathrm{IQ}\\ \mathrm{Cognitive}\;\mathrm{Composite}\;\mathrm{Score}\\ \mathrm{Detection}\;\left(\mathrm{DET}\right)\\ \mathrm{One}\;\mathrm{Card}\;\mathrm{Learning}\;\left(\mathrm{OCL}\right)\\ \mathrm{One}\;\mathrm{Back}\;\mathrm{Test}\;\left(\mathrm{ONB}\right)\\ \mathrm{Identification}\;\left(\mathrm{IDN}\right) \end{array}\right]. \end{array}$$

## 6. Experimental Results

The participants in this study were split into two cohorts for investigation. Cohort 1 was a baseline including non-patient participants and cohort 2 was a heterogeneous experimental cohort including patient and non-patient participants.

The four extracted features were combined into a least squares multiple variable linear regression model. These were regressed against each of the seven cognitive scores and indicators. The statistical outcomes of the models for cohorts 1 and 2 are outlined in Table 1 and Table 2, respectively. The detailed breakdown attribute of each of the four extracted features to the predicted cognitive scores and indicators for cohorts 1 and 2 is outlined in Table 3 and Table 4.

Owed to the significant performance of hesitation score in these multiple variable linear regression models, this score was isolated in a linear regression against each of the seven cognitive scores and indicators. The model performance and coefficient contributions of these are detailed in Table 5 and Table 6.

In published ecological studies, it is commonplace for results to present $R^{2}\in \left(0.1,0.3\right)$ and in [9] $R^{2}$ = 0.29 to $R^{2}$ = 0.38 is cited as notable to the field (among other studies referenced in Section 1). The $R^{2}$ performance of models in VStore fall in a comparable upper bound with a range, $R^{2}\in \left(0.044,0.369\right)$. Our study also expands on the approach of current literature by quantifying behavioural markers on a continuum then combining them into a multiple variable linear regression model.

### 6.1. Cognitive Scores

Participants were measured on the cognitive battery scores outlined in Section 5. The results of this study also focus on Cogstate Composite Score, the aggregate of its constituents detailed Section 3.2, age, and IQ.

### 6.2. Participant Path

Figure 3a,b show example participant paths from the non-patient and patient cohorts, respectively. The optimal route for collecting the six items that feature in the TSP landscape are mapped in red. Timestamped participant movements are mapped in gradient blue where darker mappings indicate locations where more time was spent.

### 6.3. Hesitation Score Residual Plot

**Figure 4:**
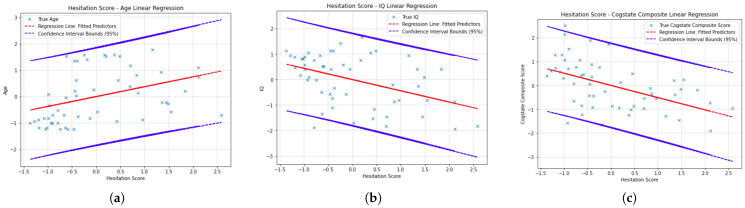
Ordinary least-squares linear regression with hesitation score at 95% confidence interval bounds against (**a**) age; (**b**) IQ; and (**c**) cogstate composite score. The red line is the regression line of fitted predictors and the crosses are the true values compared to this.

**Figure 5:**
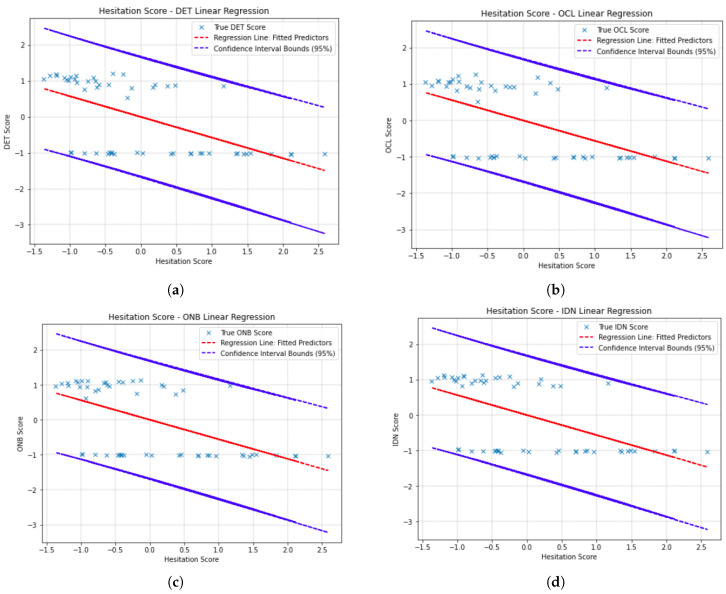
Ordinary least-squares linear regression with hesitation score score at 95% confidence interval bounds against (**a**) DET; (**b**) OCL; (**c**) ONB; and (**d**) IDN. The red line is the regression line of fitted predictors and the crosses are the true values compared to this.

Figure 4 and Figure 5 show the linear regressions for cognitive scores against hesitation score. The red line of predictors are plotted with blue crossed actual values for each participant’s scores. The blue lines enveloping these are the upper and lower bounds of the confidence interval for the regression line. There is 95% confidence that all predictor score values will fall within the upper and lower bound for the hesitation score outlined. That is to say that for each of the confidence intervals, 95% of actual cognitive composite score values fall within the confidence intervals. Detailed breakdown of the ordinary least-squares linear regression models generated via statsmodel are presented in Table 3 and Table 4.

### 6.4. Data Tables

All regressions plotted were ordinary-least-squares as the data presented a normal distribution. The R-squared values in the dataset present to which degree changes in independent variables (the four extracted features) can be attributed to the dependent variable in the study (y-predictor feature score). The F-statistic compares the linear model produced for variables against models that reduce their effect to null. Hence, presenting whether a variable is statistically significant or not. Prob(F-Statistic) informs the accuracy of the Null Hypothesis (that there is no correlation between the dependent, y-predictor score, and the independent variables, four extracted features). This expresses whether it is accurate that a variable has no effect on an outcome. P(t) expresses how likely it is that a coefficient is modelled by chance. It is essentially the gradient of the regression slope, the degree to which a change in the outcome (y-predictor feature) can be attributed to change in the input variable (four extracted features).

## 7. Discussion

In Figure 3, the participant path plot for the non-patient participant was more direct from node to node than that of the patient. Patients with psychosis visited the same locations multiple times and spent extended time at each. Both participants take complex routes at “full fat milk” as this is when the six-item TSP presents in the VR environment. The non-patient participant path is closer to the TSP solution than the psychosis patient path. The non-patient participant followed a more linear path through the environment indicative of decisive strides in their route. The patients with psychosis spent the most time at each location, captured in the hesitation score feature, following a less clearly defined path. This analysis was the first indicator of the hypothesis being correct that a behavioural difference would be notable in VR environment between patient and non-patient cohorts. A baseline homogeneous cohort, see Table 3, was used to test the computational method and features extracted before introducing a heterogeneous cohort, see Table 4, for further investigation. Age and cogstate composite score were found to present statistically significant results when modelled in a four-feature ordinary least-squares multiple variable linear regression, see Table 4. For age and IQ, the models presented $R^{2}$ = 0.38 and $R^{2}$ = 0.224, respectively. F-stat and its associated *p*-value were also statistically significant. For age this was F-Stat = 14.27 and p(F-Stat) = 14.07 ×$10^{-9}$—much smaller than 0.05, which is the threshold for which it is improbable that the feature’s coefficient is modelled by chance. With this, there is grounds to reject the Null Hypothesis—namely, that there is no correlation between the x-features in the model and the y-feature, age. For Cogstate composite, it was F-Stat = 6.713 and p(F-Stat) = 8.61 ×$10^{-5}$. In ecological studies, F-Stat ≥ 3.95 and *p*-values ≤ 0.05 are required to reject the Null Hypothesis, that there is no correlation between the independent and dependent variables. Figure 5 presents the outcomes of the linear regressions between all four extracted features and each of the cognitive score and indicator predictors. The findings from the homogeneous cohort provided the confidence in the method and features extracted to now introduce a heterogeneous cohort of patients and non-patients. All y-feature cognitive scores were found to be statistically significant when modelled on this cohort. The greatest significance was observed when the four features were modelled against the cognitive battery score constituents DET, OCL, ONB, and IDN. When considering each feature’s contribution to the model, hesitation score was most significant and in turn isolated from the other features and modelled alone against the y-features. For comparison, refer to Table 6. Figure 4 and Table 6 present the outcomes of the linear regressions between hesitation score and each of the cognitive score and indicator predictors. It is notable to observe that the statistics of F-Stat and its associated *p*-value, p(F-Stat) increased when this feature was isolated. The hesitation score found the cohort to split into two distinct groups of y-feature cognitive performance though each group had overlap in hesitation scores observed. In simplified terms this suggests that some participants with healthy cognition in the cohort hesitated as much as those presenting cognitive impairment across DET, OCL, ONB and IDN. The hesitation score alone presents a time-bound metric for performance of the task on the basis of collecting an item when it becomes visible to a participant. It is worth considering that though participants were found to take time to collect items that were visible to them, the driver for time taken may go beyond a lack of capacity in executive function. It was observed that some participants collected the items in the order they appeared if pacing up and down the shopping aisles, irrespective of when the items were first visible, systematically exploring their environment. Others hesitated most at the 6-point TSP presenting at the fridge and then swiftly followed a path close to the optimal TSP solution. The hesitation score is limited in being standardised to the 12 items in the shopping list only without considering any extra items that may have been collected. In turn, hesitation score is conservative for participants that collected extra items (captured by execution error score) or traversed additional locations where some of the items on the shopping list items may have also become visible sooner than is accounted for in the score. The further three extracted features lend context to the hesitation observed by participants by modelling optimality of path taken at a critical stage of the environment, accuracy and conflation of shopping list items, and the total distance covered in an environment with with cognitive cues (such as 6-point TSP and visible aisle headings) to devise and execute a strategy that could reduce time spent and distance travelled in completing the task. This is corroborated by the improvement in $R^{2}$ of the linear regression models when hesitation score was combined with the other three features. On Age, IQ, Cogstate Composite, DET, OCL, ONB and IDN it increased by 35%, 25%, 25%, 10%, 14%, 13% and 12%, respectively. Where $R^{2}$ presents the extent to which variation in the y-feature can be attributed to the weighting of x-features in the model. For example when hesitation score is modelled with the remaining three features in ordinary least-squares multiple linear regression, the model improves in performance. From these observations the hypotheses are validated. Cognitive impairment can be detected on the continuum it spans from patient behaviour in a VR environment. To achieve this, multiple features quantifying behavioural significance must be extracted and modelled together as each can add meaning to the other. Features can be time-bound, distance based, and measure strategy in reacting to stimuli in a VR environment. There are, however, limitations that should be considered. Firstly, use of VR is biased towards those with technological familiarity. This was accounted for in the selection process of the study but would need to be computationally accounted for should VStore be administered independent of this exclusion criteria. In future generations technological familiarity may be less relevant, at present it remains a consideration.

## 8. Conclusions

VStore fully immerses participants in a real-life scenario where symptoms would present. This computational methodology innovates on current VR assessments by quantifying behavioural presentations of cognition via four extracted features. These features can be combined into a predictive model to map extent of cognitive impairment on a continuum. VStore invites novel behavioural scoring through capture of time stamped participant movements about both translational and rotational human axes via time-bound, distance based and strategically centred features. From the observations in this study the hypotheses associated with the research questions are met. The basis of the four features extracted in this study show potential to be transferred to other VR environments and studies of cognition. This method and the features extracted strengthen potential for VR based cognitive assessments to be more sensitive and ecologically valid than current medical assessments. Extracted features are best modelled against a mathematically optimal performance, where this is not possible, to the performance of a model non-patient participant. Above all, this method shows promise for future patient studies to detect early onset of prevalent conditions associated with cognitive decline such as Alzheimer’s Disease. Future work on this proof-of-concept analysis method requires validation, as well as review by ethicists, medically qualified data scientists and relevant regulatory bodies. Future patient training sets should continue to account for demographic and technological familiarity. The application of computational methods can have an impact when understood by both the data scientists that devise them and the registered professionals that lean on them. VStore is currently being clinically trialled across the UK.

## Figures and Tables

**Table 1:** Baseline non-patient cohort results (Cohort 1) on four feature ordinary least-squares multiple variable linear regression model. Model statistics include $R^{2}$, F-statistic, and its associated *p*-value.

Feature	$R^{2}$	F-stat	*p* (F-stat)
Age	0.380	14.270	$4.07\times 10^{-9}$
IQ	0.057	1.399	0.240
Cog Comp	0.224	6.713	$8.61\times 10^{-5}$
DET	0.071	1.775	0.140
OCL	0.044	1.075	0.374
ONB	0.070	1.752	0.145
IDN	0.050	1.256	0.293

**Table 2:** Patient and non-patient cohort (Cohort 2) results on four feature ordinary least-squares multiple variable linear regression model. Model statistics include $R^{2}$, F-statistic and its associated *p*-value.

Feature	$R^{2}$	F-stat	*p* (F-stat)
Age	0.173	2.561	0.05
IQ	0.252	4.1118	0.00591
Cog Comp	0.328	5.986	0.000528
DET	0.369	7.158	0.000128
OCL	0.364	6.997	0.000154
ONB	0.359	6.872	0.000179
IDN	0.364	7.009	0.000152

**Table 3:** Baseline non-patient cohort (Cohort 1) coefficient statistics against each y predictor in four feature ordinary least-squares multiple variable linear regression model. Statistics include standard error, t-value, its *p*-value, and confidence interval boundaries (95%). Where Cst. is constant, PD is proportional distance score, RO is route optimality score, ExErr is execution error score, and H is hesitation score.

Cognitive Score	Feature	Coef	Std Error	*t*	p(t)	[0.005	0.995]
Age							
	Cst.	0.0055	0.082	0.067	0.947	−0.210	0.221
	PD	−0.2079	0.097	−2.154	0.034	−0.462	0.046
	RO	−0.0758	0.086	−0.882	0.38	−0.302	0.150
	ExErr	0.2283	0.093	2.456	0.016	−0.016	0.473
	Hes	0.6878	0.095	7.260	0.000	0.439	0.937
IQ							
	Cst.	0.0178	0.100	0.179	0.858	−0.244	0.280
	PD	0.0182	0.117	0.155	0.877	−0.290	0.326
	RO	0.0326	0.104	0.312	0.756	−0.242	0.307
	ExErr	−0.0489	0.113	−0.433	0.666	−0.346	0.248
	Hes	0.2175	0.115	1.891	0.062	−0.085	0.520
Cog Comp							
	Cst.	−0.0087	0.092	−0.095	0.925	−0.250	0.233
	PD	0.1214	0.108	1.125	0.264	−0.163	0.405
	RO	0.1457	0.096	1.515	0.133	−0.107	0.339
	ExErr	−0.1960	0.104	−1.884	0.063	−0.470	0.078
	Hes	−0.4994	0.106	−4.710	0.000	−0.778	−0.221
DET							
	Cst.	−0.0051	0.100	−0.051	0.959	−0.269	0.259
	PD	0.1101	0.118	0.933	0.353	−0.200	−0.421
	RO	0.0717	0.105	0.682	0.497	−0.205	0.348
	ExErr	0.0028	0.114	0.024	0.981	−0.296	0.302
	Hes	−0.2828	0.116	−2.439	0.017	−0.588	−0.022
OCL							
	Cst.	−0.0053	0.102	−0.052	0.958	−0.273	0.262
	PD	−0.0158	0.120	−0.132	0.895	−0.331	0.299
	RO	0.0815	0.107	0.765	0.446	−0.199	0.362
	ExErr	0.0616	0.115	0.534	0.595	−0.242	0.365
	Hes	−0.1599	0.118	−1.361	0.177	−0.469	0.149
ONB							
	Cst.	0.0002	0.101	0.002	0.998	−0.264	0.265
	PD	−0.0694	0.118	−0.587	0.558	−0.380	0.241
	RO	0.0877	0.105	0.833	0.407	−0.189	0.364
	ExErr	−0.0229	0.114	−0.201	0.841	−0.322	0.277
	Hes	−0.2101	0.116	−1.811	0.073	−0.515	0.095
IDN							
	Cst.	0.0043	0.102	0.042	0.966	−0.263	0.271
	PD	−0.2123	0.119	−1.778	0.079	−0.526	0.102
	RO	−0.0981	0.106	−0.922	0.359	−0.378	0.182
	ExErr	0.1759	0.115	1.529	0.130	−0.127	0.478
	Hes	0.0441	0.117	0.376	0.708	−0.264	0.352

**Table 4:** Patient and non-patient cohort (Cohort 2) coefficient statistics in ordinary least-squares multiple variable linear regression model against each y predictor. Statistics include, standard error, *t*-value, its *p*-value, and confidence interval boundaries (95%). Where Cst. is constant, PD is proportional distance score, RO is route optimality score, ExErr is execution error score, and H is hesitation score.

Cognitive Score	Feature	Coef	Std Error	*t*	p(t)	[0.005	0.995]
Age							
	Cst.	−0.0302	0.128	−0.237	0.814	−0.372	0.312
	PD	0.0975	0.134	0.727	0.471	−0.262	0.457
	RO	−0.1477	0.133	−1.111	0.272	−0.504	0.209
	ExErr	−0.0108	0.130	−0.083	0.934	−0.358	0.337
	Hes	0.3869	0.129	3.000	0.004	0.041	0.733
IQ							
	Cst.	−0.0249	0.123	−0.203	0.840	−0.354	0.304
	PD	0.0553	0.129	0.428	0.670	−0.291	0.304
	RO	−0.1328	0.128	−1.038	0.304	−0.476	0.210
	ExErr	−0.1719	0.125	−1.377	0.175	−0.506	0.163
	Hes	−0.4051	0.124	−3.263	0.002	−0.738	−0.072
Cog Comp							
	Cst.	−0.0011	0.118	−0.009	0.993	−0.318	0.316
	PD	−0.1767	0.124	−1.423	0.161	−0.509	0.156
	RO	0.1673	0.123	−1.359	0.180	−0.163	0.497
	ExErr	−0.1785	0.120	−1.487	0.144	−0.500	0.143
	Hes	−0.4937	0.119	−4.135	0.000	−0.814	−0.174
DET							
	Cst.	−0.0130	0.114	−0.114	0.910	−0.318	0.292
	PD	0.1073	0.120	0.896	0.375	−0.214	−0.428
	RO	−0.1295	0.119	−1.092	0.280	−0.448	0.188
	ExErr	−0.1019	0.116	−0.880	0.383	−0.412	0.208
	Hes	−0.5543	0.115	−4.815	0.000	−0.863	−0.246
OCL							
	Cst.	−0.0159	0.114	−0.139	0.890	−0.322	0.290
	PD	0.1104	0.120	0.921	0.362	−0.211	0.432
	RO	−0.1513	0.119	−1.273	0.209	−0.470	0.167
	ExErr	−0.1219	0.116	−1.051	0.298	−0.433	0.189
	Hes	−0.5335	0.115	−4.626	0.000	−0.843	−0.224
ONB							
	Cst.	−0.0142	0.115	−0.124	0.902	−0.321	0.293
	PD	0.1389	0.120	1.153	0.254	−0.184	0.426
	RO	−0.1284	0.119	−1.075	0.287	−0.448	0.192
	ExErr	−0.1131	0.116	−0.971	0.336	−0.424	0.199
	Hes	−0.5399	0.116	−4.662	0.000	−0.850	−0.230
IDN							
	Cst.	−0.0137	0.114	−0.120	0.905	−0.320	0.292
	PD	0.1235	0.120	1.028	0.309	−0.198	0.445
	RO	−0.1351	0.119	−1.135	0.262	−0.454	0.184
	ExErr	−0.1088	0.116	−0.937	0.353	−0.420	0.202
	Hes	−0.5447	0.115	−4.718	0.000	−0.854	−0.235

**Table 5:** Patient and non-patient cohort (Cohort 2) results on hesitation score ordinary least-squares linear regression model. Model statistics include $R^{2}$, F-statistic, and its associated *p*-value.

Feature	$R^{2}$	F-stat	p (F-stat)
Age	0.128	9.035	0.00407
IQ	0.201	13.09	0.000673
Cog Comp	0.262	18.47	$7.59\times 10^{-5}$
DET	0.335	26.24	$4.48\times 10^{-6}$
OCL	0.319	24.39	$8.54\times 10^{-6}$
ONB	0.318	24.22	$9.07\times 10^{-6}$
IDN	0.325	25.04	$6.80\times 10^{-6}$

**Table 6:** Patient and non-patient cohort (Cohort 2) coefficient statistics in ordinary least-squares linear regression with hesitation score against each y predictor. Statistics include, standard error, *t*-value, its *p*-value, and confidence interval boundaries (95%).

Cognitive Score	Feature	Coef	Std Error	*t*	p(t)	[0.005	0.995]
Age							
	Cst.	−0.0327	0.126	−0.260	0.796	−0.369	0.303
	Hes	0.3743	0.125	3.006	0.004	0.041	0.707
IQ							
	Cst.	−0.0229	0.123	−0.186	0.853	−0.352	0.306
	Hes	−0.4414	0.122	−3.618	0.001	−0.768	−0.115
Cog Comp							
	Cst.	0.0074	0.120	−0.061	0.951	−0.314	0.329
	Hes	−0.5116	0.119	−4.298	0.000	−0.830	−0.193
DET							
	Cst.	−0.0137	0.113	−0.121	0.904	−0.317	0.289
	Hes	−0.5756	0.112	−5.122	0.000	−0.876	−0.275
OCL							
	Cst.	−0.0163	0.114	−0.142	0.887	−0.322	0.290
	Hes	−0.5603	0.113	−4.938	0.000	−0.864	−0.257
ONB							
	Const.	−0.0155	0.115	−0.135	0.893	−0.322	0.291
	Hes	−0.5594	0.114	−4.921	0.000	−0.863	−0.255
IDN							
	Cst.	−0.0147	0.114	−0.129	0.898	−0.320	0.291
	Hes	−0.5661	0.113	−5.004	0.000	−0.869	−0.264

## Data Availability

The datasets presented in this article are not readily available due to commercial restrictions. VStore is a propriety software.
